# Fungal foraging behaviour and hyphal space exploration in micro-structured Soil Chips

**DOI:** 10.1038/s41396-020-00886-7

**Published:** 2021-01-19

**Authors:** Kristin Aleklett, Pelle Ohlsson, Martin Bengtsson, Edith C. Hammer

**Affiliations:** 1 Department of Biology, Lund University, Lund, Sweden; 2 Department of Plant Protection Biology, Swedish University of Agricultural Sciences, SLU, Alnarp, Sweden; 3 Department of Biomedical Engineering, LTH, Lund University, Lund, Sweden

**Keywords:** Microbial ecology, Fungal ecology, Fungal biology, Microbial ecology

## Abstract

How do fungi navigate through the complex microscopic maze-like structures found in the soil? Fungal behaviour, especially at the hyphal scale, is largely unknown and challenging to study in natural habitats such as the opaque soil matrix. We monitored hyphal growth behaviour and strategies of seven Basidiomycete litter decomposing species in a micro-fabricated “Soil Chip” system that simulates principal aspects of the soil pore space and its micro-spatial heterogeneity. The hyphae were faced with micrometre constrictions, sharp turns and protruding obstacles, and the species examined were found to have profoundly different responses in terms of foraging range and persistence, spatial exploration and ability to pass obstacles. Hyphal behaviour was not predictable solely based on ecological assumptions, and our results obtained a level of trait information at the hyphal scale that cannot be fully explained using classical concepts of space exploration and exploitation such as the phalanx/guerrilla strategies. Instead, we propose a multivariate trait analysis, acknowledging the complex trade-offs and microscale strategies that fungal mycelia exhibit. Our results provide novel insights about hyphal behaviour, as well as an additional understanding of fungal habitat colonisation, their foraging strategies and niche partitioning in the soil environment.

## Introduction

Fungi are fascinating organisms; Despite being classified as microbes, their mycelia can grow to form some of the largest organisms on earth [1] and their interlinked mycelial morphology can give insight into modern network theories [2]. Fungal ecology is believed to play a crucial role in shaping Earth’s ecosystems because of fungi’s ability to recycle nutrients in the system as decomposers [3], provide resources as symbionts of the majority of land plants [4], and impact the success and dynamics of other organisms as pathogens [5, 6]. All these functions ultimately depend on interactions between individual hyphal tips and their immediate environment.

Fungi live and interact with their environment mainly at the microscopic scale; their hyphae are very motile and can adjust their morphology in response to stimuli and changes in the environment [7, 8]. This type of phenotypic plasticity has also previously been used to describe plant behaviour [9, 10], particularly pertaining to plants’ ability to forage for resources [11, 12]. How hyphae respond to and interact with different types of environments at the microscopic scale still represents a significant missing piece of the puzzle in our understanding of fungal ecology.

**Fig. 1:**
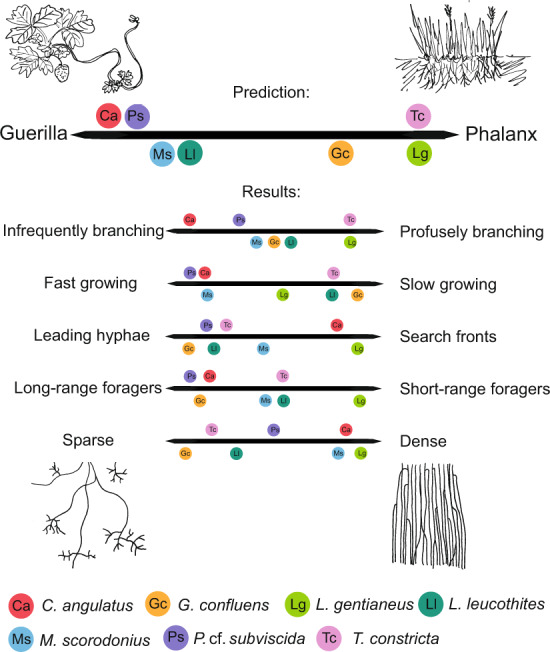
Summary of hyphal growth behaviour in the context of the ecological concept of phalanx- and guerrilla-type foraging. This concept originates from plant ecology and is used to describe growth patterns of clonal plants, where *Fragaria* (strawberry; guerrilla, left) and *Festuca* (phalanx, right) are typical examples of the concept. Guerrilla-type foraging in fungi has been defined by infrequent branching, fast growth, leading hyphae, long-range foraging and sparse growth (listed to the left), whereas phalanx-type foraging has been defined by the opposite set of characteristics: frequent branching, slow growth, a front of hyphae advancing in synchrony, short-range foraging and dense growth [15, 17]. The seven examined species were hypothetically placed along the continuum of the phalanx and guerrilla division based on assumptions associated with what type of litter the different species have been found to grow on (Table S1) (above), and further placed along the continuums of their defining trait components based on the results of this study (below). Based on our results, none of the examined species fell clearly into one of the categories, or even at a comparable location within the continuum for all trait axes. Instead, we saw that species could be typical guerrilla for one trait and typical phalanx for another.

Inspiration to describe fungal growth patterns at the mycelial scale can be drawn from plant ecology, especially from the descriptions of clonal plant growth. The different ways in which clonal plants colonise new territory has been conceptualised as *guerrilla* and *phalanx* growth forms [13, 14]—terms that can also be used to describe fungal mycelial growth patterns [15–17] (Fig. 1). A guerrilla growth form fungus is characterised by opportunistic, far-reaching explorative hyphae; a short lifespan; and low competitive ability [15]—a beneficial strategy in a patchy, ephemeral resource landscape [17]. In contrast, a phalanx growth form fungus colonises new territories by short-range foraging, growing dense mats of hyphae and advancing slowly as a united front [15]. This strategy would be expected among specialists that exploit more complex and long-lasting substrates by investing in the production of large amounts of extra-cellular enzymes to break them down and antibiotics to fend off competitors [17]. Mycelia may also switch between phalanx- and guerrilla-type growth. For example, finding a nutrient-rich path could induce profuse branching and transition the fungi from guerrilla to phalanx growth form [17]. Understanding how fungal foraging strategies differ between species is important for being able to draw conclusions about their co-existence and spatial niche partitioning in the environment [18].

The fungal filamentous growth form is well adapted to the heterogeneity of the soil habitat [19]. The soil pore space constitutes a 3D labyrinth of channels and hidden corners that the fungi need to navigate [20] and organic matter—the most important nutrient source for soil microorganisms—is very patchily and ephemerally distributed in the soil. Soil is impenetrable to light, preventing direct visual observation, and therefore little is known about how fungal hyphae behave in their natural environment. In experimental settings, fungi are often cultured and propagated on structurally homogenous, typically nutrient-rich media in which the effect of small-scale structural heterogeneity on fungi’s ability to locate, consume, store and transport nutrients in the soil may be misjudged. Studying fungi in their natural soil substrate is notoriously difficult and involves slicing the colonised soil to later reconstruct spatial context [21, 22]. To assess ecological questions concerning how fungi would grow in their natural habitat, alternative experimental systems have therefore been designed and utilised e.g. the surface of compacted soil [17], porous ceramic plates [23, 24] and 3D printed soil proxies [25]. However, these systems still suffer from being opaque or nonadjustable in terms of spatial structure at the microscale. Recently, microfabrication techniques called microfluidics, previously used mainly in biomedical applications, have been applied to simulate soil pore structures [26]. The unique abilities of these systems to confine single cells and manipulate minute concentrations of liquids [27] make them ideal for simulating the microscale heterogeneity of soil systems [28]. Microfluidic chips have proven useful to study fungal hyphae [29–31], and can be applied to address a wide range of questions in soil ecology [32].

In this study, we explore fungal growth behaviours in a newly developed experimental chip system which allows for manipulation of micrometre-scale structural heterogeneity, while simultaneously facilitating real-time imaging of hyphal behaviour. We used microfluidic techniques to develop a chip that strives to simulate soil heterogeneity with the help of geometric pore space models that allow us to compare hyphal behaviour for a multitude of fungal species. We refer to our experimental system as “Soil Chips”, and the specific design developed for this study as “the Obstacle Chip”.

By studying hyphal behaviour in the Obstacle Chip, we were able to examine species differences among a set of seven different soil fungi, all broadly classified in the same functional group of litter decomposers. We hypothesised that the hyphal behaviour would be predictable based on ecological variables such as what type of litter the different species are adapted to grow on. We predicted that the species would express either a guerrilla- or a phalanx-like foraging behaviour (Fig. 1) based on which type of substrate it is known to grow on and observations of growth rates in plate cultures (Table S1). We then examined variables that could help ascertain this inside the chip at a hyphal level.

**Fig. 2:**
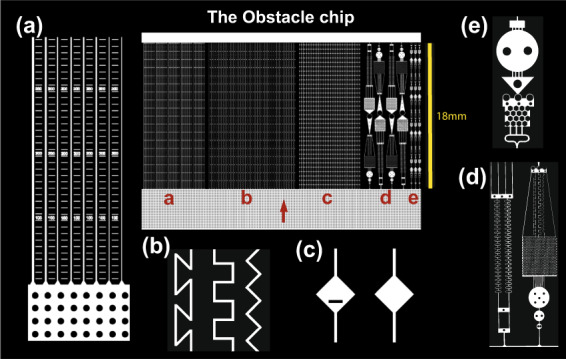
Schematic overview of the design of the Obstacle Chip. The chip design is shown in the centre, surrounded by enlarged details from the different experiments (**a**–**e**). The red arrow shows the hyphal growth direction. **a** Parallel straight channels in a series of different widths (20, 15, 10, 8, 6, 4 µm; *n* = 6) with each width repeated five times within the chip. Rulers were incorporated between the channels to measure how far the hyphae reached under microscope. **b** Channels of 10 µm width angled in a *zigzag* pattern with 90° corners, meandering *square* pattern with 90° corners or a *z-shaped* pattern with 135° corners, organised in a randomised order, *n* = 11. **c** Channels of 10 µm width with the repeated occurrence of 140-µm-diameter diamond-shaped openings that either was free for passage, included a 50-µm-wide and 10-µm-thick obstacle blocking the straight passage of the fungi, or a random occurrence of open and blocked openings in the same channel in randomised order, *n* = 12. **d** Larger obstacle courses with a combination of challenging structures for the fungi to navigate through. **e** Smaller-sized obstacle courses with more frequent repetition of obstacles.

We designed geometrical microstructures to address the following questions (see corresponding figures in Fig. 2a–e): (a) How does hyphal growth respond to a restriction of radial expansion? (b) How is hyphal growth influenced by forced turns through increasingly wider angles—do turns wider than 90° impede growth? (c) Do hyphae explore sudden openings of a pore space after long paths of constriction and do obstacles induce branching? (d) and (e) How do fungal hyphae behave in a complex obstacle course? Do fungi have the same spatial preferences if structures are presented in a complex arrangement? We hypothesised that a classical phalanx-strategist would be impeded by lateral restriction and wide angles, and explore spaces by frequent branching, whereas a classical guerrilla strategist would be less affected by restriction and better able to navigate through complex structures. For each species, we studied hyphal growth responses in a nutrient-void space and explored whether there were structures that fungal hyphae commonly do not pass—potentially creating physically inaccessible parts of the pore space labyrinth.

## Materials and methods

### Chip design

In the Obstacle Chip, we created a micro-structured environment to test the growth behaviours and limitations of fungal hyphae and lined them with rulers to measure how far and how fast the hyphae were advancing inside the channels. We included four different types of structures for four spatially confined, independent experiments inside the chip (Fig. 2). The first section (a) had straight channels of six different widths (20, 15, 10, 8, 6 and 4 µm). Each chip contained five sets of replicates with the six different widths. The second section (b) had 10-µm-wide, angled channels with three different types of corners that the hyphae had to navigate past, either: *zigzag* (90° corners, diverting 45° from the original growth direction), meandering *square* (90° corners, diverting 0° or 90° from the original growth direction) and *z-shaped* channels (135° corners, diverting 0° or 135° from the original growth direction and forcing the fungi to repeatedly turn back towards the hyphal front in order to advance), *n* = 11, randomised using a custom script provided by UrbanLISP (http://www.urbanlisp.com). The third section (c) had 10-µm-wide straight channels, with repeated diamond-shaped openings, increasing the width of the channel to 140 µm. Each channel (*n* = 12) contained a total of 33 openings, interconnected by 400-µm-long narrow sections. To be able to measure whether these sudden openings could alter fungal growth behaviour or induce branching, one-third (12) of the channels contained openings without obstacles, one-third (12) of these channels also included a 50-µm-wide and 10-µm-thick obstacle inside the diamond-shaped openings, and one-third (12) of the channels had a randomised mix of open and obstacle-blocked openings along the channel. The final section of the chip contained two types of obstacle courses (d) and (e) where multiple kinds of channel types and obstacles are combined at two different scales (e contained smaller shapes than d). The original chip design contains an additional section with a honey-comb-patterned pillar system that was not evaluated in this study and therefore is not included in figures to avoid confusion. Chips were fabricated in polydimethylsiloxane (PDMS) and glass using methods of photolithography previously described in [32]. For details regarding the specific settings used in this experiment, see Supplementary information (SI).

### Fungal cultures and colonisation of the chips

The following species were grown in the chips for examination: *Coprinellus angulatus* (Peck) Redhead, Vilgalys & Moncalvo*, Psilocybe* cf. *subviscida* (Peck) Kauffman*, Gymnopus confluens* (Pers.) Antonín, Halling & Noordel*, Tricholomella constricta* (Fr.) Zerova*, Leucopaxillus gentianeus* (Quél.) Kotl*, Mycetinis scorodonius* (Fr.) A.W. Wilson & Desjardin, and *Leucoagaricus leucothites* (Vittad.) Wasser. The species chosen are all sequenced Basidiomycete members of the order Agaricales within the eco-functional group of litter decomposers (for more information see Table S1).

Fungal cultures were grown on malt medium agar plates (malt extract broth, Oxoid, Unipath Ltd, Hampshire, England). To inoculate the chips, equal size 1-cm-wide and 6.5-cm-long (the length of the chip) rectangular pieces were cut from the hyphal fronts of the seven species, catching the hyphal front of the mycelium with a buffer of unexplored media in front of it. Plugs were placed upside down in contact with the chip, lining the entire entrance of the chips, and with the growth direction of the hyphal front pointing into the chip. Autoclaved pieces of gauze soaked in deionised water were placed adjacent to the chips inside the Petri dishes, which were then sealed with plastic paraffin film (Parafilm Bemis NA) to retain the moisture. Because of the different growth speed and morphology of the fungal cultures, optimum times for when to cut cultures and inoculate chips were established for each species (Table S1). This means that cultures were of different ages when introduced to the chip design, but comparable in their colony life-cycle stage as they were all in an active growth stage, and colonies were big enough to obtain enough material for placing a full fungal front along the entrance of the chip. No water or growth medium was injected into the chips, and the chips’ structures remained dry throughout the experiments. The environment inside the chips was maintained dry by the use of an agar plug instead of liquid for inoculation, the presence of the pillar-filled entry system with a comparatively low level of capillary forces, and the use of dry chips with predominantly hydrophobic surface properties. However, for the two species that were cultured in 26 °C, there was some condensation noted inside the chip at the early stages of colonisation. The only probable source of nutrients within the system was what the hyphae themselves could transport from the initial inoculation plug of malt medium. Microcosms were then kept dark at a temperature of 20 °C (except *L. leucothites* and *M. scorodonius* which were kept at 26 °C) for the duration of the study. We tested whether hyphae grow differently when another part of their mycelium encounters different structures (using the whole coherent chip vs cutting it into pieces of the single experiments) for *Psilocybe* cf. *subviscida*, but no difference in growth distance and speed was detected. However, this idea could warrant further investigation across more species and configurations.

### Measurements and microscopy

**Fig. 3:**
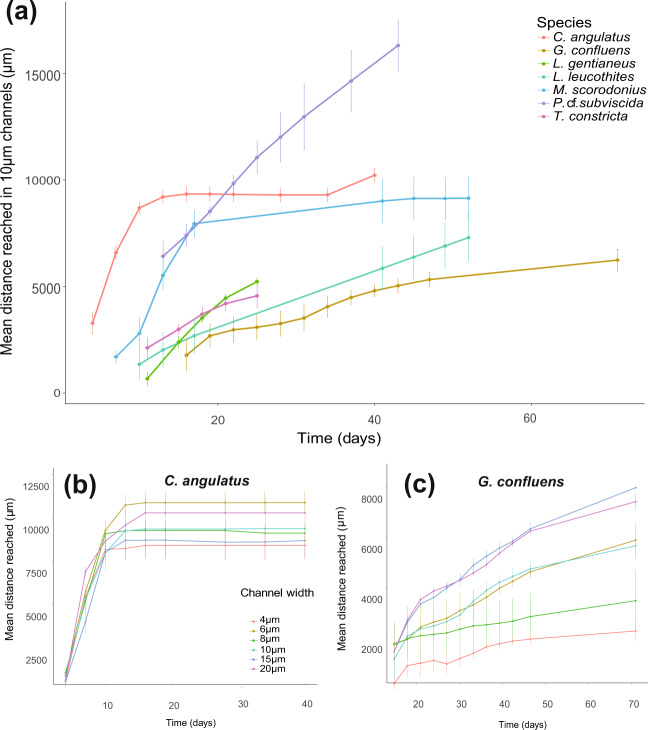
Hyphal growth rates by the fungal species over time—measured in the section of the chip with straight channels of different widths (Fig. 2a). **a** Comparison of the mean growth rate of the different species in colonised 10-µm-wide straight channels, *n* = 6. **b** Comparison of mean growth rates in straight channels of different width (20, 15, 10, 8, 6 and 4 µm) for *C. angulatus*, which did not show a significant difference in advancement between different widths (*p* = 0.52), **c** similar comparison for *G. confluens* that advanced significantly shorter distances in narrower channels (*p* < 0.0001). Error bars show ±1 SEM.

The growth speed of the different fungi was recorded in 3-day intervals until growth ceased (see Fig. 3), by measuring the distance the furthest hyphal tip had reached. Final measurements of the maximum distance reached by hyphae, and branching patterns inside the chip were recorded after all hyphae had ceased to grow inside the chip. Hyphae in an opening (Fig. 2c) were classified as “branched” if at least one of the hyphae present had branched.

Photos and all visual measurements were made using an inverted microscope (Nikon DIAPHOT 300) by the same person (KA) throughout the sampling.

### Statistics

Data were statistically evaluated in JMP Pro 13.0 (SAS Institute Inc., Cary, USA). Hyphal speed and colonisation rates in different channel widths were analysed using a univariate repeated measures analysis of variance (ANOVA) model. The colonisation of differently angled channel shapes was analysed with a one-way ANOVA followed by a Tukey–Kramer HSD posthoc test. Differences in colonisation between straight and angled channels were not statistically evaluated because of different spatial locations of the testing arenas within the chip. Multivariate principal component analysis (PCA) was performed on all seven species showing correlations of the measured trait variables listed in Table S2. If data existed for two or more related measures, which were thematically related and autocorrelated, only one of those was kept in the analysis. This was the case for the following traits, and the first alternative was used in the analysis: “branching in open diamonds” and “branching in blocked diamonds”; “far in angled channel” for z-shaped, square-shaped and zigzag channels; “far in small obstacle course a” and “b”. Results are presented as mean ± SEM.

## Results

All seven fungal species examined in this experiment successfully colonised the microfluidic chips’ air-filled pore spaces. Species differed strongly in their strategies to explore the chip space, varying in growth speed, mycelial density, branching patterns, and maximum distance covered. When comparing the advancement of hyphae inside 10-µm-wide straight channels over time, we found that the tested species showed considerable variation in colonisation patterns (Fig. 3a). Mean maximal growth distances varied from 5 mm to completion of the whole channel (18 mm). Some hyphae of *C. angulatus* and *P*. cf. *subvicida* reached the end of the channel, preventing us from determining the true maximum distance that they were able to explore. the highest growth speed (tracking the furthest-reaching hyphae in channels) over a 3-day period was seen in *C. angulatus and M. scorodonius*, which grew with speeds of 1.7 and 0.98 mm/day respectively. Hyphae of *G. confluens* and *L. leucothites*, continued to actively grow in the chip for more than 2 months and ceased advancement in the middle of the chips, while still reaching a maximum pace of single hyphae of 0.82 and 0.54 mm/day. *L. gentianeus* and *T. constricta* both had a slowly advancing hyphal front (max speed of the fastest recorded hyphae 0.53 and 0.61 mm/day, respectively), and a low maximum proliferation into the chip. However, the two species strongly differed in mycelial morphology, while *T. constricta* slowly advanced with a few solitary hyphae, *L. gentianeus* produced a large number of parallel hyphae at the hyphal front which layered on top of each other in the channels, making it hard to determine the exact number of hyphae growing in each channel. Fast-growing species advanced with relatively low numbers of typically 5–20 for *C. angulatus*, or about 1–3 hyphal tips per channel for *P*. cf. *subviscida*. In particular, *C. angulatus* often showed thigmotropism (growing along the walls of the channels).

### Lateral restriction of hyphae

Growth speed (the extension rate of the fastest-growing hyphae in the channels) was compared in six different channel widths (4–20 µm) to test the effect of spatial constriction on hyphal growth, maximum reach, and growth speed over time (Fig. 3a). While most species were not significantly affected by the tested channel widths (e.g., *C. angulatus* Fig. 3b), *G. confluens* grew better in increasingly wider channels (*p* < 0.0001; Fig. 3c), and growth of *P*.cf. *subvicida* was especially impeded in the narrowest, 4-µm-wide channels (*p* < 0.0001; Fig. S1).

### Turning angles

**Fig. 4:**
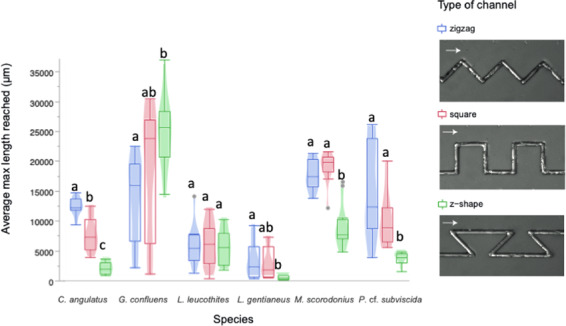
Distances reached by the fungal species within the differently angled channels (Fig. 2b). Box plots show the mean final distances reached by the hyphae in channels of the same type (*n* = 11). Different letters indicate a significant difference of colonisation distances for each species according to ANOVA followed by Tukey-Kramer’s HSD test at *p* < 0.05 (Table S3). The legend contains examples of the differently angled channels colonised by *P*. cf. *subviscida*, where arrows indicate growth directions.

We compared how far the hyphae extended in differently angled channels (Fig. 4), and results showed that several of the species were significantly hindered in their reach by having to turn around corners sharper than 90° (z-shaped channels) (*C. angulatus* *p* < 0.0001, *L. gentianeus* *p* = 0.001, *M. scorodonius* *p* < 0.0001, *P*. cf. *subviscida* *p* < 0.0001, Table S3). Only *C. angulatus* grew significantly further in the zigzag channels than in the other two channel types (Fig. 4). *G. confluens*, in contrast to all other species, grew furthest in z-shaped channels (*p* = 0.015). Both *G. confluens* and *P*. cf. *subviscida* showed a large variation in distances grown inside the angled channels, and the latter occasionally grew to the end of the angled channels. Comparing the maximum distances reached inside these three types of channels (Fig. 4) to the distances covered in the straight channels (Fig. 3), most fungi reached shorter distances in angled than in the straight channels. *M. scorodonius* and *G. confluens were* clear exceptions, the former reaching approximately double the distance in zigzag and square channels compared to straight ones, and the latter more than four times the distance in z-shaped channels compared to straight ones.

### Branching as a response to obstacles and exploration of sudden openings

**Fig. 5:**
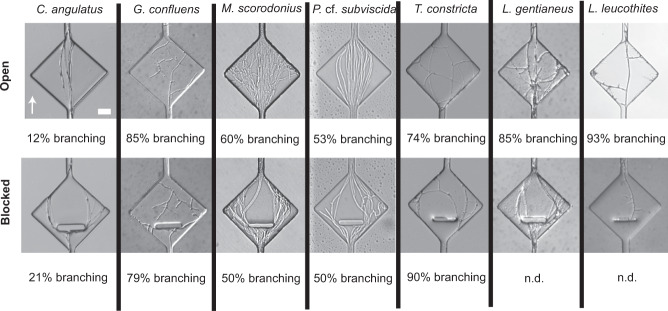
Hyphal growth responses to sudden channel openings (with or without a perpendicular obstacle) (Fig. 2c) in the different species examined. The level of branching was recorded for colonised openings in each chip and listed as a percentage for each type of widening (open and blocked). If the opening was colonised by multiple hyphae, the occurrence of one branching hypha was enough to record it as ‘branching’. The percentage of branching was calculated per number of colonised openings. Percentage of branching could not be determined (n.d.) for blocked openings of *L. gentianeus* and *L. leucothites* since their hyphae frequently grew beneath the blockages. The arrow indicating growth direction and the scale bar of 20 µm apply to all images.

Examining fungal responses to sudden openings in the environment by diverging or branching of hyphae, we found that hyphae commonly explored the open spaces by branching. *C. angulatus* stood out among the species examined by having a very low level of branching in both blocked (21%) and open (12%) diamonds (Fig. 5). On the other end of the spectrum, *T. constricta* was found to branch in as many as 90% of all blocked diamonds and 74% of open ones (Fig. 5). Numerical data for blocked diamonds are missing for *L. gentianeus* and *L. leucothites* because of suboptimal obstacle fabrication in combination with strong tip force, resulting in hyphae regularly growing straight beneath the obstacles, breaking the bond of the chip. However, both species showed frequent branching in the un-blocked openings (*L. gentianeus* 85%, and *L. leucothites* 93%). For *L. leucothites*, the branching inside open diamonds happened differently compared to some of the other species, not immediately as the hyphae entered the opening, but successively over time as small lateral branches started to form from an older leading hypha. The number of openings that the fungi colonised differed between species, thus these data are presented as a percentage of the colonised openings. The branching frequency was more consistent within species (both open and blocked diamonds) than among species examined, indicating specific and consistent space exploration patterns of the examined species, rather than evidence for a consistent pattern of obstacle-induced branching (Fig. 5).

### Hyphal plasticity and navigation in complex structures

**Fig. 6:**
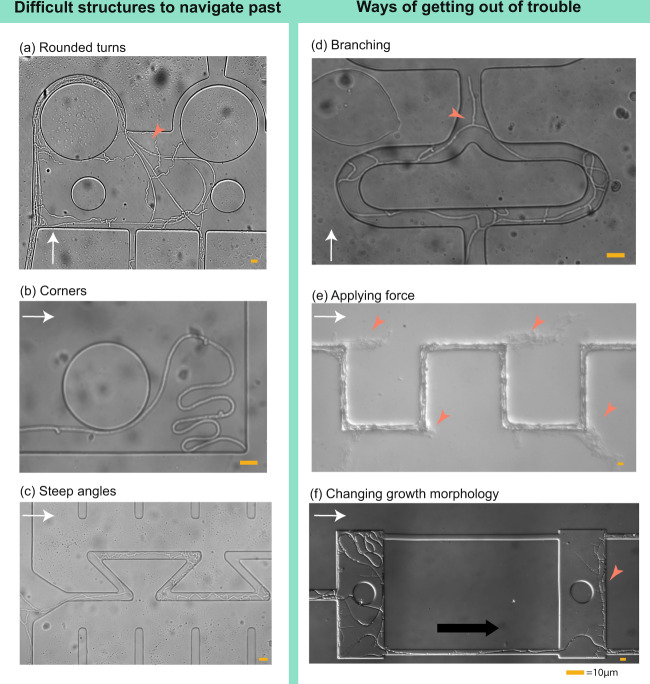
Fungal navigation in complex structures. Certain structures in the chips proved notoriously difficult for fungal hyphae to navigate past (**a**–**c**), and different growth strategies were applied by the fungi to increasing their foraging range past these obstacles (**d**–**f**). **a** Rounded turns confused the growth direction of hyphae (here *P*. cf. *subviscidae)* and led them to grow back towards their origin (red arrowhead). **b** Corner-trapped hyphae (here *G. confluens)*. The tip was not able to navigate out of the corner, and the hyphae instead elongated behind the tip in a folding manner. **c** Sharp angles restricted hyphal advancement of many species (as seen in Fig. 3, here *P*. cf. *subviscidae*) and in some cases led hyphae to turn back towards their original growth direction. **d** Branching increased the likelihood that a newly formed tip found the passage for progression (red arrowhead). In that case, the apex switched to this tip (here *P*. cf. *subviscidae)*. **e** Hyphae hitting solid chip parts could apply tip force. Some species (here *L. gentianeus*) were able to break through the bonding of the chip and continue to grow between the PDMS and the glass slide in its original direction (red arrowheads), instead of following the maze pattern. **f** *G. confluens* altered its growth morphology from thicker, flexible runner hyphae (young growth morphology, left red arrowhead) to thinner hyphae with frequent lateral short branches (old growth morphology, right red arrowhead). The white arrows indicate growth direction.

The seven litter decomposers compared in this study showed a large variation in growth patterns when faced with obstacle courses—a complex assembly of channel shapes, angles, and arrangements, some which were also studied separately in other sections of the chip (Fig. 2d, e). From these data, we identified structures that were generally difficult for fungi to grow through: round structures leading the hypha back towards the original inoculum (Fig. 6a), corners (Fig. 6b), and sharp angles of 135° (Fig. 6c). We documented three principal strategies commonly applied by the fungi for navigation through difficult spatial structures (illustrated in Fig. 6d–f): I. Branching to switch the lead hypha—this strategy, in which the fungi branched and continued to grow mainly from the newly formed hyphal tip, proved efficient both for getting past obstacles, getting out of corners and re-finding the original growth direction of the hyphal front (Fig. 6d); II. Applying force—fungi with rigid hyphae could get past obstacles by simply pushing the hyphae in between the glass and the bonded PDMS-layer of the chip, or through the PDMS (Fig. 6e); III. Mycelial plasticity—this behaviour was observed specifically for hyphae of *G. confluens*, which over time changed growth strategy drastically in terms of hyphal thickness, wall smoothness, branching frequency and angles (Fig. 6f).

**Fig. 7:**
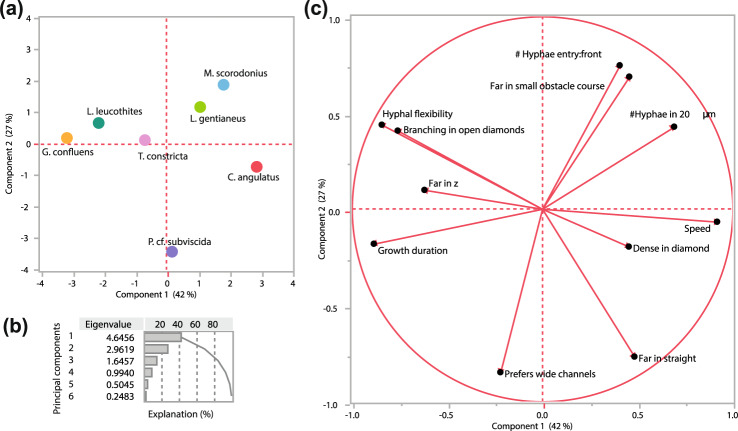
Principal component analysis (PCA) of the measured traits for the seven fungal species investigated (Table S2). **a** Score plot for the seven species along two dimensions of PC1 and PC2. **b** Explanation of the variation. Cumulatively explained variation of PC1 and PC2 is 69.4%. **c** Loading plot of the variables included in the PC analysis. Dashed lines connect the different locations of the loading variables for distance growth in different spatial structures. Details on the measurements can be found in Table S2.

Differences and similarities between the species were visualised as a PCA (Fig. 7a–c) based on the measured trait variables (Table S2). The analysis identified *G. confluens* and *C. angulata* as most different in their growth strategies (Fig. 7a, distant placing along PC1). The loading plot visualises the trade-offs between different traits and growth patterns among the tested species, such as growth speed versus maximum reached distance or dense colonisation of the openings versus hyphal flexibility and branching frequency (Fig. 7c). Interestingly, distance measurements performed in different spatial settings clustered in opposing areas of the loading plot: The vectors for “growth far in z-shaped channels” and “growth far in straight channels” point to opposite ends along PC1, and are almost uncorrelated to the factor “growth far in the complex obstacle course”, which instead clusters along PC2 (Fig. 7c).

### Mycelial differentiation, exudations and reproductive behaviour in mature cultures

**Fig. 8:**
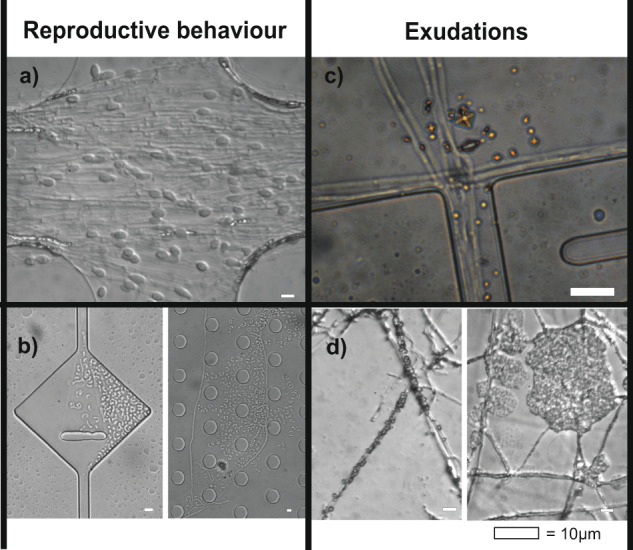
Observations of reproductive behaviour and exudations inside the Obstacle Chips. **a** *L. gentianeus* forming chlamydospores in the entrance system of the chip. Chlamydospores have previously been documented in *L. gentianeus* [49]. **b** *P*. cf. *subviscida* forming monokaryotic arthroconidia from dikaryotic hyphae. *P*. cf*. subviscida* has previously been shown to produce monokaryotic arthroconidia [50, 51]. **c** *P*. cf. *subviscida* exudates forming copper coloured crystals (likely oxalates [52]). **d** *L. leucothites* exudates forming crystals that cluster around the hyphae.

There were several fungal species that, towards the end of the experiment, started to reproduce asexually by forming spores or conidia (Fig. 8a, b). There was likely an increasing accumulation of organic compounds inside the chips, mainly due to fungal exudates. This habitat modification caused by the fungi themselves is likely species-dependent and would be an interesting topic for future investigation. In chips colonised by *P*. cf. *subviscida*, we found signs of hyphal exudates in the form of loose octahedral-shaped crystals, and amber-coloured droplets in mature cultures (Fig. 8c). In *L. leucothites* chips, we also saw crystals, but smaller and more densely aggregated at the surface of the hyphae (Fig. 8d).

## Discussion

The Obstacle chip has given us new insight into the differences in behaviour found at the level of single hyphae, even among fungi broadly classified as having a similar lifestyle as litter decomposers. Our results open a discussion about the commonalities and differences in general fungal foraging behaviour, and whether there are fungal behaviours unique to species or groups of species. Are there fungi that have developed more efficient ways to navigate the microscopic mazes of the soil, and could some hyphal tip behaviours be more beneficial in specific environments or situations?

### Trade-offs in space exploration patterns

We initially hypothesised that the species would express either phalanx (e.g., *L. gentianeus*) or guerrilla (e.g., *G. confluens*) type growth (Fig. 1), in line with the recent large species comparison on Petri plates [33] which found that fast-growing species exploit spaces, while slow-growing species tend to explore. However, when looking more closely at the five distinguishing characteristics for the phalanx- and guerrilla-continuum identified in the literature (branching frequency, growth speed, mycelial expansion patterns, foraging range and mycelial density) [17], none of our species fit unambiguously into one of the categories. Several species were even clearly categorised as guerrilla-type for some attributes, and phalanx-type for other (*C. angulatus, T. constricta, M. scorodonius, L. leucothites*) (Fig. 1).

The microstructures inside the chip allowed us to investigate fungal foraging strategies and behaviour in much greater detail than was previously possible, and identify trade-offs in hyphal foraging through multivariate trait analyses (Fig. 7). Some of the traits are included in the distinction of phalanx- and guerrilla-type foraging (e.g. the trade-off between growth speed and branching), while other trait differences reflected the microscale patterns that we were investigating for the first time. Most striking was how growth distances were affected by spatial structures where species grew well either in undisturbed paths or paths with different kinds of obstacles, leading to much further growth in some structures than in others. The species showing the starkest contrasts in this respect were *C. angulatus* and *G. confluens*, where the former grew as quick and far as possible, while the latter grew much longer distances when structural obstacles needed to be passed. We expect that those strategies would be successful for different types of foraging (Table S1): *C. angulatus* is commonly found in burnt areas on charcoal debris and thus is specialised in opportunistic exploitation of easily available resources and relief from competition [34, 35], while *G. confluens* is a litter decayer of hardwood and coniferous needles [36] and should therefore be more adapted to withstand competition while exploiting resources with heavy exoenzyme investments. *L. gentianeus* is also known to grow on needles from coniferous trees [37], but is a dense-growing phalanx-type forager. Our results suggest that *G. confluens* may be a more thorough forager of smaller food patches than *L. gentianeus. L. gentianeus* probably contributes more in later degradation stages, consumes higher quantities, and is more broadly distributed—a situation for which the slow but continuous growth with a meticulous spatial scanning of a large volume may be most efficient. The hyphal foraging behaviour of these two species during starvation demonstrates a clear niche separation, but could look different when they encounter a nutrient source. To investigate foraging strategies in more depth we suggest developing chip systems that also include full nutrient supply, different nutritional conditions such as foraging rewarded with patchy nutrient sources, and multispecies settings (competition) to unravel the microscale effects of chemical heterogeneity and social interactions on fungal behavioural niches and hyphal decision making. Hyphal-scale investigations should be an important future complement to large-scale mycelial trait studies such as the recent one by Maynard et al. [38].

### Hyphal behaviour, polarity and directional memory

It has been discussed whether hyphae could possess directional memory, steered by its Spitzenkörper [29], guiding it to trail along walls (thigmotropism) [30], and used to navigate through complex labyrinths [39] much like the porous soil system. Studies of polarity in fungi have suggested that species may differ in their sense of polarity (maintaining their growth direction), where for example *Clitocybe nebularis* that form so-called ‘fairy rings’ would be a good example of a species that show a strong sense of polarity (demonstrated nicely by cutting out pieces of mycelium and attempting to change their direction of growth [40]). A recent publication [41] showed results of whole mycelia having preferential growth in the direction in which it had previously encountered a food source, suggesting a certain level of memory being retained at the level of the whole mycelium. Previous studies in micro-chips have shown that single hyphae of *Neuraspora crassa* and *Pycnoporus cinnabarinus* only reluctantly diverge from their original growth direction when forced through cornered passages [39, 42, 43], whereas other species like *Armillaria mellea* are less inclined to maintain its original growth direction [42]. Held et al., therefore, suggested that certain fungi could possess a stronger directional memory than other species [42], and Hanson et al. claimed that *Pycnoporus cinnabarinus* hyphal apices would not turn around corners if the resulting growth direction diverged more than 93–94° from the initial growth direction, but instead fold up in bundles [43].

In our chip, we challenged the different species by providing channels with bends sharper than 90° (135°-wide turns in z-shaped channels), forcing the hyphae to turn back towards its original growth direction. Our results from both the z-shaped channels and additional observations in the obstacle courses showed that all of the species examined were capable of turning in angles of 135°, even though several species (*C. angulatus, L. gentianeus, M. scorodonius, P. subviscida*) were significantly hampered in their proliferation inside channels that forced them to turn back and against the initial growth direction (Fig. 4). Our dataset indicates that the rigidity of a specie’s hypha determines whether it is able to make sharp bends, and we suggest that the location of the Spitzenkörper in relation to a more or less stiff hyphal wall could be a mechanical explanation of polarity and differences in directional memory between species. If the hyphal growth was directed around rounded obstacles, we also found that the initial growth direction of the mycelium was lost, and the hyphae would in most cases continue to grow back continuously towards its origin instead of re-adjusting to its initial direction and advancing further into the labyrinth of the chip (Fig. 6a). This could be explained by the slower gradual deviation from the growth direction not causing as much friction against a stiff cell wall. One way that fungi to retained its growth direction after travelling along curved paths, or in the z-shaped channels, was to abandon apical dominance and grow a lateral branch towards the open pore space, which after a while would take over as lead hyphae (Fig. 6d). This behaviour could be triggered by changes in internal-hyphal or inter-mycelial chemical gradients, on which more research is needed. However, this way of progression is not very efficient as it produces many “dead-end-hyphae” in the mycelium pointing back towards the centre of the culture.

### Restrictions to fungal growth and strategies for getting out of trouble

The Obstacle Chip simulates principal aspects of the microscale soil pore space environment, and we were able to examine whether certain geometries remain inaccessible and unexplored in the system. The structures that we identified as impeding continuous hyphal growth (sharp turns in channels, sudden openings creating lee-like unexplored areas behind hyphal tips, trapping corners) also occur, in principle, in real soil pore spaces [19, 44]. Studies from these types of systems could therefore help us understand whether fungal hyphae are spatially restricted in the soil, and potentially explain stabilisation processes of organic matter in the soil through physical disconnection [45].

In contrast to previous studies by Harris et al. [20] and Otten et al. [21], we did not find preferential growth of hyphae in wider pore spaces, though we examined it at a finer spatial scale. However, hyphal growth might change the connectivity within a pore space system, by either filling up space—e.g. when a flexible hypha curled up (Fig. 6b, c), thus blocking passage for other hyphae or organisms—or by pushing their way through structures (Fig. 6e), depending on the rigidity of the specie’s hyphal wall. This force could be used to push into soil aggregates and access occluded organic matter. The protrusive forces of tip-growing cells of both pollen tubes and oomycete hyphae have been quantified in microfluidic devices [46, 47]—a method which could also be used to screen the penetrative ability of a larger number of fungal species. Not only soil environments constitute complex spatial challenges to fungi. Host tissues, such as plant and animal organs—where hyphae commonly grow along inter-cellular spaces [48], are also difficult to study in real life. Microscale studies of hyphal behaviour and tropisms inside microfluidic chips simulating these types of host cell structures could significantly extend our understanding of fungal pathogenic and mutualistic associations.

## Conclusions

We show that there are broad differences in fungal growth and space exploration strategies between species, and that the fungi encounter different spatial challenges in micrometre geometries. At the hyphal scale, the classifications of guerrilla- and phalanx-type foraging did not conclusively explain the growth patterns and foraging behaviours seen, but the Obstacle Chip revealed a multidimensional trait assembly that could better explain niche separation and co-existence of fungi in ecosystems. We see Soil Chips as an important new tool for answering many emerging questions at the frontier of soil science, fungal ecology and ecophysiology, with the possibility to provide us with a better understanding of fungal behaviour, the dynamics of soil carbon decomposition, mycelial network resource allocation and a chance to connect microbial community data to function.

## Supplementary information

Supplementary information
