# Supplementary material for: Fungal foraging behaviour and hyphal space exploration in micro-structured Soil Chips
Source: ISME J. 2021 Jan 19;15(6):1782–93. doi: 10.1038/s41396-020-00886-7 (PMC8163874; doi:10.1038/s41396-020-00886-7)
Supplement: Supplementary file 1 — Supplementary information [file 41396_2020_886_MOESM1_ESM.pdf]

SUPPORTING INFORMATION

Methods for fabrication of the Obstacle chip:

The design of the chip was drawn in AutoCAD 2015 software (Autodesk), and printed as a glass photolithography mask (JD photo data, UK: http://www.jd-photodata.co.uk/). The chip was fabricated using polydimethylsiloxane (PDMS) moulding on a photoresist master generated by UV lithography [33]. A thick negative photoresist, SU-8 5 (MicroChem Corp, USA), was spin-coated onto a glass plate using at 1250 rpm for 60 seconds, resulting in a layer thickness of approximately 7 µm. After a soft bake step (90 °C for 5 minutes on a hot plate) the resist was exposed with a MA4 Karl-Suss mask aligner to define the pattern in the photoresist, followed by a post-exposure bake step. The pattern was developed in mr-Dev 600 (MicroChem) for 3 minutes and then rinsed using isopropanol (VWR International). PDMS (Sylgard 184, Dow Corning, USA) was prepared with a base-to-curing agent ratio of 10:1 following the protocol from the manufacturer, thoroughly stirred and poured on top of the master. Subsequently, the PDMS-covered master was then degassed in a vacuum chamber at -25kPa for approximately one hour. Finally, the PDMS was cured in an oven at 60°C for two hours after which the cured structure could be peeled off from the master.

The pieces with the desired pattern imprint were carefully cut out from the PDMS and bonded to glass slides by exposing surfaces of both to UV light in an oxygen plasma chamber (UV Ozone Cleaner – ProCleaner™, Bioforce Nanosciences) for 10 minutes and subsequently pressing the two surfaces together. To achieve optimal bonding, glass slides were cleaned with acetone, 75% ethanol, and deionized water and air-blown dry before use. Bonded chips were then placed in sterile petri dishes of 140 mm diameter.

Supporting Table S1. Culturing conditions and background ecology of the fungal strains and species used in the experiment.

Strain	Order	Species	Temp. (ºC)	Inoculation age (days)	Ecology
**175,51**	Agaricales	*Coprinellus angulatus*	20	7	Grows after fire events, attached to small fragments of charcoal, early coloniser (Lange & Smith, Alexander 1953; Hernández-Rodríguez *et al.* 2013)
**406,79**	Agaricales	*Gymnopus confluens*	20	36	Grows on hardwood duff and leaf litter (Hughes & Petersen 2015)
**146,42**	Agaricales	*Leucoagaricus leucothites*	26	15	Grows in grassy areas or on disturbed grounds (Vellinga & Davis 2006).
**437,85**	Agaricales	*Leucopaxillus gentianeus*	20	36	Grows on conifer litter, rarely on deciduous litter (Garnier-Delcourt *et al.* 2011)
**850,87**	Agaricales	*Mycetinis scorodonius*	26	15	Grows on coniferous needles or dead deciduous leaves in forests and heaths (Petersen & Hughes 2017)
**101986**	Agaricales	*Psilocybe* cf. *subviscida*	20	15	Grows on raw humus of grasses, straw and partly digested dung (Boekhout *et al.* 2002)
**660,87**	Agaricales	*Tricholomella constricta*	20	36	Grows in pastures on hardwood litter, is slightly nitrophilous (Kalamees 1992; Hofstetter *et al.* 2014)

Supporting Table S2. Response variables measured for the seven examined Basidiomycetes that were included into the principal component analysis (Fig. 6). "Mean speed" was measured as the first derivative of the growth curve. "Growth duration" was measured as the time hyphal growth inside the chip was observed. "Far in straight channels" denotes the mean maximum distance the hyphae reached in straight channels of 10µm width. "Far in z-shaped channels" denotes the mean maximum distance the hyphae reached in z-shaped channels, 10µm width, meandering taken into account. "Branching in open diamonds" is the percentage of openings without a perpendicular obstacle in which at least one hypha branched. "Dense in diamonds" is the percentage of the hyphal coverage of openings without a perpendicular obstacle. "Hyphal flexibility" was measured as the angle an hypha formed when hitting the obstacle in perpendicularly blocked openings, where 90 denotes greatest bendability and 45 highest rigidity. "Far in small obstacle course" denotes the mean maximum distance the hyphae reached in an obstacle course with complex and irregular shapes, relative measure. "#Hyphae entry:front" denotes the ratio of the number of hyphae at the beginning of the 20 µm wide channels (200 µm for the entry) to the number of hyphae at the mycelial front (100 µm from the outermost tip).

Species	Mean speed [µm*d-1]	Growth duration [d]	Far in straight channels [µm]	#Hyphae in 20µm channels	Far in z-shaped channels [µm]	Branching in open diamonds [%]	Dense in diamonds [%]	Prefers wide channels	Hyphal flexibility	Far in small obstacle course	#Hyphae entry:front
C. angulatus	928	11	15000	15	2500	12	20	1.0	58	5.1	2.4
M. scorodonius	722	26	9000	10	8000	60	80	1.0	68	10.7	9.8
G. confluens	62	90	5500	2.5	24500	85	16	2.9	85	4.2	2.1
P. cf. subvicida	352	60	18500	1	4000	53	75	5.7	57	1.9	1
L. gentianeus	342	35	6500	20	500	85	40	1.2	63	5.1	6.1
T. constricta	227	27	4500	2	0	74	30	1.3	71	3	2
L. leucothites	173	70	8000	3.5	6200	93	10	1.0	88	3.7	3.5

Supporting Table S3. One-way ANOVA of the raw data for how far hyphae of the different species grew into channels of different shapes and angles.

Species	Channel shape	F ratio	p-value	Tukey-Kramer's HSD
C. angulatus	all	80.79	<0.0001	
	Zigzag			A
	Square			B
	z-shape			C
G. confluens	all	4.9205	0.0147	
	Zigzag			A
	Square			AB
	z-shape			B
L. leucothites	all	0.0366	0.96	
	Zigzag			A
	Square			A
	z-shape			A
L. gentianeus	all	4.19	0.0241	
	Zigzag			A
	Square			AB
	z-shape			B
M. scorodonius	all	41.26	<0.0001	
	Zigzag			A
	Square			A
	z-shape			B
P. cf. *subviscida*	all	13.00	<0.0001	
	Zigzag			A
	Square			A
	z-shape			B

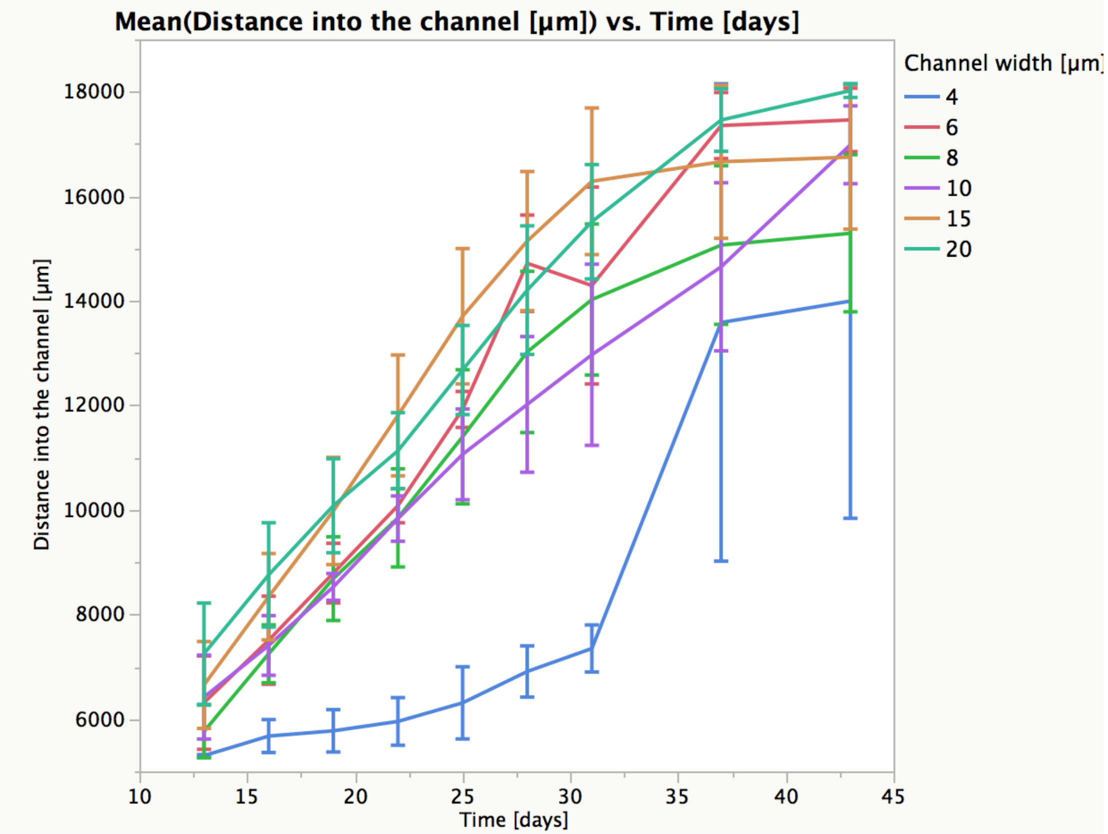

Supporting Figure S1. Comparison of hyphal growth of *P.*cf. *subvicida* over time in straight channels of different widths (4-20µm).

References SI

Boekhout, T., Stalpers, J., Verduin, S.J.W., Rademaker, J. & Noordeloos, M.E. (2002). Experimental taxonomic studies in Psilocybe sect. Psilocybe. *Mycol. Res.*, 106, 1251–1261.

Garnier-Delcourt, M., Reckinger, C., Tholl, M.-T. & Turk, J. (2011). Notes mycologiques luxembourgeoises. IV. *Bull. Soc. Nat. luxemb*, 112, 39–50.

Hernández-Rodríguez, M., Oria-de-Rueda, J.A. & Martín-Pinto, P. (2013). Post-fire fungal succession in a Mediterranean ecosystem dominated by Cistus ladanifer L. *For. Ecol. Manage.*, 289, 48–57.

Hofstetter, V., Redhead, S.A., Kauff, F., Moncalvo, J.-M., Matheny, P.B. & Vilgalys, R. (2014). Taxonomic Revision and Examination of Ecological Transitions of the Lyophyllaceae (Basidiomycota, Agaricales) Based on a Multigene Phylogeny. *Cryptogam. Mycol.*, 35, 399–425.

Hughes, K.W. & Petersen, R.H. (2015). Transatlantic disjunction in fleshy fungi III: Gymnopus confluens. *MycoKeys*, 9, 37–63.

Kalamees, K. (1992). Tricholomella, a new genus, with the distribution data of Tricholomellaconstrictum, comb. nov. in East Europe and Asia. *Persoonia*, 14, 445–447.

Lange, M. & Smith, Alexander, H. (1953). The coprinus ephemerus group. *Mycologia*, 45, 747–780.

Pantidou, M., Watling R. & Gonou Z. (1983). Mycelial characters, anamorphs, and teleomorphs in genera and species of various families of Agaricales in culture. Mycotaxon 17, 409–432.

Petersen, R.H. & Hughes, K.W. (2017). An investigation on Mycetinis (Euagarics, Basidiomycota). *MycoKeys*, 24, 1–138.

Valenzuela, E. & Garnica, S. (2000). Pseudohelicomyces , a new anamorph of Psilocybe, 104, 738–741.

Vellinga, E.C. & Davis, R.M. (2006). Lepiotaceous fungi in California, U.S.A. – 1., 98, 197–204.
